# An Investigation of the Factor Structure of the Self-Compassion Scale

**DOI:** 10.1007/s12671-017-0803-1

**Published:** 2017-09-30

**Authors:** Seonaid Cleare, Andrew Gumley, Chris J. Cleare, Rory C. O’Connor

**Affiliations:** 1 Suicidal Behaviour Research Laboratory, Institute of Health and Wellbeing, College of Medical, Veterinary and Life Sciences, University of Glasgow, Gartnavel Royal Hospital, 1055 Great Western Road, Glasgow, G12 0XH UK; 2 Aberfeldy, Scotland UK

**Keywords:** Self-compassion scale, Factor analysis, Factor structure, Bi-factorial, Omega

## Abstract

The Self-Compassion Scale (SCS) is the most widely used measure of self-compassion. The scale is constructed of six factors measuring positive and negative components of compassion. Support for this factor structure has been subject to debate and alternative factor structures have been proposed. We tested the proposed factor structures against existing models of the SCS including one derived from an exploratory factor analysis of our data. Respondents (*n* = 526) completed the original version of the SCS online at two time points, at baseline (time 1) and 2.5 months later (*n* = 332, time 2). Exploratory factor analysis (EFA) was carried out on time 1 data and confirmatory factor analyses (CFA) were conducted on time 2 data and retested using time 1 data. The EFA yielded a five-factor model. CFA was used to compare the following models: Neff’s original six-factor correlated and higher-order models; a single-factor, two-factor, five-factor model (as suggested by the EFA) and a bi-factorial model. The bi-factorial model was the best fit to the data followed by the six-factor correlated model. Omega indices were calculated and yielded support for the bi-factorial model of SCS. In conclusion, this study supports the use of the six-factor scoring method of the SCS and the use of an overarching self-compassion score.

## Introduction

The importance of self-compassion has long been recognised in Buddhist and Eastern philosophical traditions but, only recently, has its importance as a research construct distinct from other psychological constructs such as mindfulness (Kuyken et al. 2010) or self-esteem (Neff and Vonk 2009) been acknowledged. This has led to considerable growth in research examining the role of self-compassion particularly in the aetiology of both physical and mental well-being (Barnard and Curry 2011). Although researchers such as Gilbert (2009) have suggested definitions of self-compassion, one of the most widely used definitions is that put forward by Neff (2003b) who conceptualised self-compassion as follows:

Being touched by and open to one’s own suffering, not avoiding or disconnecting from it, generating the desire to alleviate one’s suffering and to heal oneself with kindness. Self-compassion also involves offering non-judgmental understanding to one’s pain, inadequacies and failures, so that one’s experience is seen as part of the larger human experience. (Neff 2003b, p. 87)

Within this definition, Neff conceptualised self-compassion as being composed of the following three components:

(a) self-kindness – extending kindness and understanding to oneself in instances of perceived inadequacy or suffering rather than harsh judgment and self-criticism, (b) common humanity – seeing one’s experiences as part of the larger human experience rather than seeing them as separating and isolating, and (c) mindfulness – holding one’s painful thoughts and feelings in balanced awareness rather than over-identifying with them in an exaggerated manner (Neff and Lamb 2009, p. 864)

Evidence for a link between self-compassion and mental well-being is increasing (for review see Barnard and Curry 2011). What is more, enhancing self-compassion may also have physical health benefits (Hall et al. 2013). Self-compassion has been shown to be a more accurate predictor of overall well-being than self-esteem (Neff and Vonk 2009) and it accounted for additional variance in anxiety and depression beyond that explained by self-esteem (Gilbert 2009). Self-compassion may protect against emotional distress. In a recent meta-analysis, MacBeth and Gumley (2012) found an association between self-compassion and lower levels of depression, anxiety and stress. Although the majority of studies were cross-sectional, the findings suggested that greater self-compassion was associated with mental well-being and that self-compassion may be associated with a reduction in some forms of emotional distress.

The main assessment tool used was the Self-Compassion Scale (SCS; Neff 2003a). Concerns have been raised that by measuring ‘negative’ components of compassion; specifically that the SCS is measuring self-criticism, rumination and social isolation, rather than self-compassion (MacBeth and Gumley 2012; Muris 2015). In a more recent meta-analysis, Muris and Petrocchi (2017) found that as the total score includes the negative components, then it might lead to an overestimation of the relationship with symptoms of psychopathology as the negative components are more strongly associated with psychopathology (*r* = .47 to –.50) than the positive components (*r* = − .27 to − .34). Neff (2016), however, described self-compassion as requiring an interaction between the positive and negative components of compassion and, as a consequence, she developed the SCS to assess compassion as per her definition (Neff 2003b).

According to Neff (2003a, b, 2016), the SCS has a six-factor structure with three positive and three opposing negative components that are interconnected. Specifically, the SCS assesses the following: (1) self-kindness: a person’s acceptance of personal flaws and ability to self-soothe in times of distress versus (2) self-judgement: expressions of self-critical or judgemental beliefs; (3) common humanity: the recognition of personal shortcomings as something that everyone experiences versus (4) feelings of isolation: feeling alone in their faults and (5) mindfulness: maintaining a non-judgemental awareness of thoughts and emotions versus (6.) over-identification with thoughts: becoming overwhelmed and wrapped up in emotions or thoughts. A series of confirmatory factor analyses were then used to evaluate the model fit. These showed that a six-factor correlated model was an ‘adequate fit’ to the data (Non-normed fit index (NNFI) = 0.90; Comparative Fit Index (CFI) = 0.91; Neff 2003a, b) and a higher-order model (NNFI = 0.88, CFI = 0.90) was also proposed as a reasonable fit (Neff 2003a, b) and was initially used to support the use of the SCS to give a total self-compassion score.

Since its original publication, the factor structure of the SCS has received considerable attention: studies have yielded mixed findings with some authors reporting support for the six-factor correlated model (Azizi et al. 2013; Castilho et al. 2015; Garcia-Campayo et al. 2014; Lee and Lee 2010; Mantzios et al. 2013) whereas other studies have been unable to replicate this factor solution (López et al. 2015; Petrocchi et al. 2013; Williams et al. 2014). Support for the higher-order model has been more sparse with only a few studies reporting it a fit to their data (Castilho et al. 2015; Cunha et al. 2016; Dundas et al. 2016).

As a result, many authors have proposed alternative factor structures which have included a single-factor model (i.e. an overarching single self-compassion construct; Deniz et al. 2008) and a four factor model where the positive factors are correlated and there is a distinct general negative factor (Zeng et al. 2016). The most widely proposed model is a two-factor solution comprised of self-compassion (total of the positive items) and self-coldness (total of the negative items; Gilbert et al. 2011). This solution has also been found when the SCS has been administered in Dutch (López et al. 2015) and Portuguese (Costa et al. 2015). Indeed, the majority of independent studies into the SCS have been carried out cross-culturally with researchers translating the scale into Greek (Mantzios et al. 2013), Iranian (Azizi et al. 2013), and Spanish (Garcia-Campayo et al. 2014) and evaluating the model fit of the adapted scales. This has led to some problems with translating the scale. López et al. (2015), for example, had to omit two of the items (self-kindness subscale item 5, ‘I try to be loving towards myself when I’m feeling emotional pain’; self-judgement subscale item 21, ‘I can be a bit cold-hearted towards myself when I'm experiencing suffering’) as the items did not translate into Dutch. This is not an uncommon occurrence as items are worded to suit the culture they are developed in and translation can change the context and meaning of items (Auer et al. 2000; Behling and Law 2000). It is not surprising, therefore, that adapting the scale for use in other cultures may slightly alter what is being measured which could affect item/factor loadings.

The incongruity in the factor structures found by previous researchers may suggest that the factor structure of the SCS is not stable and would benefit from further robust analyses. Indeed, Neff (2016) suggested that the higher-order structure may not be the most appropriate conceptualisation of compassion. Furthermore, recent studies (e.g. Neff et al. 2017; Tóth-Király et al. 2016) have investigated the factor structure further via alternatives to higher-order models and instead added a bi-factorial component alongside the six factors in the SCS model. Bi-factorial modelling assesses covariance between factors that arises from the presence of an overarching factor (in this case, self-compassion), whilst allowing the individual factors to retain and account for variance in their own subset of items (Reise et al. 2010).

Neff et al. (2017) found evidence supporting the six-factor correlated model in both non-clinical and clinical populations. In the non-clinical populations, the bi-factorial model was a comparable fit to the six-factor solution; however, it did not improve the model fit. Consequently, the authors suggested that further research using bi-factorial modelling was warranted. Since Neff (2016) suggested a bi-factorial model might best fit the measurement of self-compassion, several studies have employed this analysis using translated versions of the SCS in French, Brazilian Portuguese and Hungarian (Kotsou and Leys 2016; de Souza and Hutz 2016; Tóth-Király et al. 2016). For example, Tóth-Király et al. (2016) investigated the six-factor correlated and bi-factorial models using the Hungarian version of the SCS. The researchers compared model fit of the six-factor correlated model and the bi-factorial model using confirmatory factor analysis (CFA) and exploratory structural equation modelling (ESEM; combination of exploratory and confirmatory factor analysis techniques) and found that when using the CFA, neither model was an adequate fit to their data, but when using ESEM, both models fitted the data with the bi-factorial model being the best fit to the data. Although the focus on translated versions of the scale is welcome, there have been no independent replications of the bi-factorial model using the English language version of the scale.

With this in mind, the present study aimed to independently investigate the factor structure of the English language version of the SCS using both exploratory and confirmatory factor analytic techniques. Confirmatory factor analysis was used to compare the fit of the emergent exploratory factor structure to the alternative models described in the extant literature including the six-factor correlated model and the higher-order model (Neff 2003a, b) and the bi-factorial model proposed by Neff et al. (2017). An exploratory factor analysis was employed to explore if there was an alternative model that was a better fit to our data.

## Method

### Participants

Five hundred twenty-six adults completed the SCS at time 1 (t1). Participants were aged between 16 and 64 years (M = 23 years old, SD = 5.4). Three quarters of the sample (76%; *n* = 405) were female, and the sample was predominantly White (90%, *n* = 473). Sixty-three per cent (*n* = 332/526) of participants completed the SCS at time 2 (t2), 2.5 months later. The mean age for the t2 sample was 24 years old and primarily female (*N* = 249, 75%) and 92% identified themselves as White.

### Procedure

This study employed a prospective design. Ethical approval was granted by the University of Glasgow College of Medical, Veterinary & Life Sciences Ethics committee. Participants were recruited by convenience sampling methods. These included emails sent to students and information about the study being shared on social media. The email explained the purpose of the study and included a link to the online survey. The link took potential participants to the full study information page. To ensure informed consent, all participants actively selected that they had consented to take part in the study before being able to proceed to the questions. Participants completed the SCS at both time points allowing the stability of self-compassion to be explored across 2.5 months.

### Measures

#### Self-Compassion Scale

The SCS is a 26-item measure assessing the components of self-compassion: self-kindness versus self-judgement (e.g. ‘I try to be loving towards myself when I’m feeling emotional pain’ vs. ‘I’m disapproving and judgmental about my own flaws and inadequacies’); common humanity versus feelings of isolation (e.g. ‘When things are going badly for me, I see the difficulties as part of life that everyone goes through’ vs. ‘When I think about my inadequacies, it tends to make me feel more separate and cut off from the rest of the world’) and mindfulness versus over-identification with thoughts (e.g. ‘When something upsets me I try to keep my emotions in balance’ vs. ‘When I’m feeling down I tend to obsess and fixate on everything that’s wrong’). Items are scored 1 (almost never) to 5 (almost always) on a Likert-type scale. The scale is most often used to either give an overall compassion score, or to show how someone scores on the individual subscales. When calculating an overall self-compassion score, the three negative components of compassion are reverse scored, but the items are not reversed when calculating subscale scores.

Test-retest coefficients for the subscales were moderately correlated and ranged from *r* = .66 to .88. In the present study the total SCS was found to have excellent internal consistency (time 1 Cronbach’s *α* = .92, time 2 *α* = .95). For both time points, internal consistency (see Table 4 for full details) for the subscales ranged from fair (mindfulness subscale showing the lowest internal consistency) to good. Test-retest reliability was established as good for both the overall scale (*r* = .87, *P* < 0.01, *α* = .93) and the subscales (range *α* = .80–.89).

### Data Analyses

There are two main forms of factor analysis: exploratory and confirmatory factor analysis. Exploratory factor analysis (EFA) is a data-driven process primarily used in the development of questionnaires. In EFA, the researcher does not specify the factor structure, allowing related variables to cluster, thus creating factors (Child 1990). Comrey and Lee (1992) suggested using the following cut-offs to assess item loadings: 0.32 poor, 0.45 fair, 0.55 good, 0.63 very good and 0.71 excellent. CFA is used to further test hypotheses about the internal structure of a measure. In CFA, the researcher specifies the model parameters (i.e. number of factors, which variables load on to each factor) a priori and uses CFA to determine how well the data fit to the parameters. CFA is also important in establishing a scale’s internal consistency (Albright and Park 2009). The EFA was conducted on the t1 data using SPSS version 22 and the CFAs were carried out using AMOS graphics (version 22).

#### Missing Data

Participants who had completed fewer than 21 items of the scale items (80%) were classified as incomplete and their data were omitted from the analysis (*n* = 162). Following exclusion of the latter, at both time points, 0.08% of participants had missing data on between 1 and 4 items. A missing value analysis established that there was no pattern to the items missed (t1 *χ* ^2^ = 427.27, DF = 436, *P* = 6.08; t2 *χ* ^2^ = 420.786, DF = 435, *P* = 0.679), and as a result, the missing data were replaced using expectation-maximisation replacement methods.

Prior to conducting the factor analysis, the data were screened for any variables that were highly correlated to each other (*r* > .9) and potentially indistinguishable from other items (multicollinearity): no variables were found to be correlated over the 0.9 threshold. The sample’s sufficiency for factor analysis was also assessed using the Kaiser-Meyer-Olkin (KMO) measure of sampling adequacy. This ranges from 0 to 1 and Tabachnick and Fidell (2013) suggested that scores over 0.6 suggest suitability for factor analysis. The KMO for the sample was very good (KMO = 0.93) and Bartlett’s test of sphericity was significant (*χ* ^2^ (325) = 5944.3, *P* < 0.05). All the items correlated with at least one other item at a 0.3 level, further supporting the data’s suitability for factor analysis. Review of the diagonals on the anti-image correlations showed that they were all over 0.5 so no items were removed prior to analysis.

The data were assessed for outliers; across both time points, 14 univariate and two multivariate cases were found. All analyses were run including and excluding these cases and there were no differences in the results, so the cases were included in the analyses reported here.

#### Exploratory Factor Analysis

The EFA was carried out using Costello and Osborne’s (2005) guidelines for best practice for EFA; the maximum-likelihood method with oblique rotation (direct oblimin) was selected for the EFA as it allowed for the factors to be related.

#### Confirmatory Factory Analysis

In keeping with the maximum-likelihood method that we employed in the EFA, we assessed the model fit on the Comparative Fit Index (CFI), Tucker-Lewis Index (TLI), standardised root mean square residual (SRMR) and root mean square error of approximation (RMSEA). We did not rely upon chi-square as it has been found to be too sensitive to sample sizes in excess of 250 (Bentler and Bonett 1980). There is some debate over which cut-offs should be used for the RMSEA to indicate a good model fit. MacCallum et al. (1996) suggested that 0.08–0.10 shows a mediocre fit, and below 0.08 shows a good fit, although Steiger (2007) has since suggested 0.07 as the cut-off for a good fitting model. There is greater consensus regarding TLI and CFI scores, with 0.90 indicating an acceptable fit, and a score of over 0.95 indicating a good fit (Hu and Bentler 1999). A SRMR value < 0.08 is considered a good fit (Hu and Bentler 1999). The Akaike information criterion (AIC) was also used to compare the fit of different models; the model which has the lowest AIC value indicates the best fit to the data. The omega indices were calculated using the Omega software (Watkins 2013) for the bi-factorial model to estimate the reliability of the overarching self-compassion factor when all variance from the latent factors is removed (Brunner et al. 2012). This index provides useful information about whether the scores from a specific factor can be interpreted with confidence or if only the total score should be used.

In order to replicate Neff et al.’s (2017) study, CFA was used to evaluate the fit of the following series of models: the (1) higher-order model, (2) the six-factor correlated model originally proposed by Neff (2003a, b), (3) the single-factor model, (4) the two-factor model consisting of self-coldness and self-compassion factors (Gilbert et al. 2011), (5) the bi-factorial model testing if the SCS consists of a general self-compassion factor and six specific factors (Neff et al. 2017) and finally (6) the five-factor correlated model extracted by EFA from our t1 data.

## Results

Six hundred and ninety-eight people started the online survey. Those who did not complete the self-compassion measure (*n* = 162) were excluded from the main analyses. This yielded a sample of 526 adults who completed the SCS at time 1 (t1). Chi-square tests showed that there were no significant differences on demographic variables between those who completed the SCS at both time points and those who only completed the SCS at baseline. The *t* tests revealed no differences between the EFA (t1) and the CFA (t2) samples in age or in any of subscales of the SCS. The majority of participants reported no experience of meditation or mindfulness (t1 *N* = 391 (74%); t2 *N* = 262 (79%)); of those who reported engaging in meditative practices, only 20–23% of people reported practising at least every couple of months.

As shown in Table 1, all of the SCS subscales and total score were all significantly inter-correlated, as anticipated. The subscales were moderately to highly correlated. Common humanity showed the lowest associations with the three negative subscales (self-judgement *r* = − .33, perceived isolation *r* = − .39 and over-identification with thoughts *r* = − .38). The SCS total score was most strongly correlated with the self-kindness (*r* = .81) and self-judgement (*r* = − .82) subscales.

**Table 1:** Correlations between subscales and SCS total score

	CH	MFN	SJ	ISO	OID	SCS total
SK	.464	.630	− .673	− .449	− .462	− .805
CH	–	.584	− .333	− .389	− .381	− .666
MFN	–	–	− .457	− .446	− .529	− .769
SJ	–	–	–	.614	− .602	− .819
ISO	–	–	–	–	− .651	− .772
OID				–	–	− .783
SCS total					–	–

*P* < 0.05

*SK* self-kindness, *SJ* self-judgement, *CH* common humanity, *ISO* perceived isolation, *MFN* mindfulness, *OID* over-identification

### Exploratory Factor Analysis

The EFA revealed a potential five-factor model with all factors having eigenvalues over 1 and these cumulatively explained 49% of the variance. Parallel analysis (PA) was used to confirm the factor retention. PA is a recommended procedure to establish factor retention (Courtney 2013; O’Connor 2000). PA was conducted using the syntax available from O’Connor’s website (people.ok.ubc.ca/brioconn/nfactors/nfactors.html).

PA creates correlation matrices by generating random variables and data sets based on the number of variables and sample size of the actual data. The average eigenvalues from the computed correlation matrices are then compared to the eigenvalues from the real data correlation matrix. Factors from the real data can be retained as long as they are greater than the mean eigenvalue generated from the random data matrices. As this was the case for all of our 5 factors, we retained the EFA model.

An examination of the item loadings between factors showed two items (i.e. with correlations over 0.3) cross-loaded on more than one factor. Item 4 (‘When I think about my inadequacies, it tends to make me feel more separate and cut off from the rest of the world’) loaded on factors 1 and 5 and item 14 (‘When something painful happens I try to take a balanced view of the situation’) loaded on to both factors 2 and 3. As these were the only problematic items, they were retained in the analysis on the factors they had loaded the highest on. Table 2 below shows the EFA loadings and factor structure. There were a few items that had lower loadings (around 0.32 level; Tabachnick and Fidell’s (2013) guidance on the lowest cut-off for factor loadings) on their respective factors, but these were not viewed as problematic as they were distributed across the scale rather than clustered on a single factor.

**Table 2:** Factor loadings from exploratory factor analysis

Item	Factor 1—self-criticism	Original subscale	Factor loading
1	I’m disapproving and judgmental about my own flaws and inadequacies.	SJ	.807
2	When I’m feeling down I tend to obsess and fixate on everything that’s wrong.	OID	.517
6	When I fail at something important to me I become consumed by feelings of inadequacy.	OID	.571
8	When times are really difficult, I tend to be tough on myself.	SJ	.542
11	I’m intolerant and impatient towards those aspects of my personality I don’t like.	SJ	.474
16	When I see aspects of myself that I don’t like, I get down on myself.	SJ	.576
23	I’m tolerant of my own flaws and inadequacies.	SK	.628
26	I try to be understanding and patient towards those aspects of my personality I don’t like.	SK	.378
	Factor 2—balance/acceptance		
3	When things are going badly for me, I see the difficulties as part of life that everyone goes through.	CH	.516
7	When I’m down and out, I remind myself that there are lots of other people in the world feeling like I am.	CH	.801
10	When I feel inadequate in some way, I try to remind myself that feelings of inadequacy are shared by most people.	CH	769
14	When something painful happens I try to take a balanced view of the situation.*	MFN	.322
15	I try to see my failings as part of the human condition.	CH	.500
17	When I fail at something important to me I try to keep things in perspective.	MFN	.343
	Factor 3—emotional reactivity/emotion dysregulation		
9	When something upsets me I try to keep my emotions in balance.	MFN	.453
20	When something upsets me I get carried away with my feelings.	OID	.723
24	When something painful happens I tend to blow the incident out of proportion.	OID	.693
	Factor 4—self-kindness		
5	I try to be loving towards myself when I’m feeling emotional pain.	SK	.604
12	When I’m going through a very hard time, I give myself the caring and tenderness I need.	SK	.833
19	I’m kind to myself when I’m experiencing suffering.	SK	.833
21	I can be a bit cold-hearted towards myself when I’m experiencing suffering.	SJ	− .353
22	When I’m feeling down I try to approach my feelings with curiosity and openness.	MFN	.404
	Factor 5—isolation		
4	When I think about my inadequacies, it tends to make me feel more separate and cut off from the rest of the world.**	ISO	.391
13	When I’m feeling down, I tend to feel like most other people are probably happier than I am.	ISO	.465
18	When I’m really struggling, I tend to feel like other people must be having an easier time of it.	ISO	.448
25	When I fail at something that’s important to me, I tend to feel alone in my failure.	ISO	.460

*Cross-loaded to factor 3 .310, **Cross-loaded to factor 1 .346

### Confirmatory Factor Analysis

Confirmatory factor analyses were run on both the t2 and t1 data and the following series of models reported in Neff et al.’s (2017) study were evaluated: a single compassion factor, a hierarchical model of compassion (Neff 2003a), the six-factor correlated model (Neff 2003a), the two-factor ‘self-compassion and self-coldness’ model (Gilbert et al. 2011) and the bi-factorial model of self-compassion. In addition to these, we conducted CFA on the five-factor model that emerged from our EFA. Fit statistics for the different factor models are shown in Table 3.

**Table 3:** Model fit time 1 and time 2

Model	CFI	TLI	SRMR	RMSEA	AIC	*χ* ^2^ (DF)
Time 1 data (*N* = 526)						
Single-factor	.71	.68	.08	.09	2143.9	1987.9 (299)
2-factor	.79	.77	.07	.09	1675.7	1517.7 (298)
5-factor	.85	.84	.07	.07	1299.1	1123.1 (289)
Higher-order	.84	.82	.07	.08	1407.0	1239.0 (293)
6-factor	.88	.86	.06	.05	1183.8	997.8 (284)
*Bi-factorial* ^*a*^	*.91*	*.88*	*.06*	*.05*	*1023.9*	*787.9* (*259*)
Time 2 data (*N* = 332)						
Single-factor	.77	.75	.08	.11	1673.9	1466.8 (299)
2-factor	.85	.84	.07	.09	1218.3	1060.3 (298)
5-factor	.88	.87	.07	.08	1003.6	879.6 (289)
Higher-order	.88	.87	.08	.08	1045.2	877.2 (293)
6-factor	.92	.91	.05	.06	852.23	666.2 (284)
*Bi* *-* *factorial* ^*a*^	*.95*	*.94*	*.06*	*.05*	*757.67*	*521.7* (*259*)

^*a*^ *Best* *fit* *to* *the* *data* t1 = CFA run using time 1 data; t2 = CFA run using time 2 data

Using the cut-offs for the fit criteria mentioned above (CFI and TLI > 0.9, SRMR < 0.08, RMSEA < 0.07), it is clear that the single-factor model did not fit the data nor did the two-factor model (self-compassion and self-coldness items). Examination of the five-factor model showed that although the model was approaching an adequate fit to the data, it did not fulfil the fit criteria. This was the same for the higher-order model. The six-factor correlated model was a good fit for the data with all the items loading on their respective factors well (ranging from good 0.55 to excellent 0.86, see Table 5).

The six-factor correlated model was characterised by all the factors being moderately to highly inter-correlated. Applying Cohen’s (1988) cut-offs, the correlations ranged from moderate (0.3) to very highly correlated (e.g. perceived isolation and over-identification having the highest correlation at 0.94). The bi-factorial model was also fitted to establish whether there was an overarching self-compassion factor in addition to the six factors. As shown in Table 5, when the overarching self-compassion factor was included in the t2 data, the factor loadings for the majority of the items remained high and all remained above .32 suggesting they loaded well on the self-compassion factor. When the same model was run using the t1 data, items 18 (‘When I’m really struggling, I tend to feel like other people must be having an easier time of it’) and 20 (‘When something upsets me I get carried away with my feelings’) loaded poorly on the self-compassion factor (.28 and .26, respectively).

The inclusion of the overarching self-compassion factor significantly reduced the variance shared by the factors, and as shown in Table 3, this improved the model fit across all measurement criteria and improved the AIC from 852.23 in the six-factor model to 757.67. CFA’s using the t1 data revealed a similar pattern; this time, however, none of the models fully fitted all of our criteria for a good model fit as the TLI for the bi-factorial model dropped to under 0.9, but this model remained the closest fit to the data.

Omega indices (*ω* and ωH) were calculated for the bi-factorial model (Table 4) to assess the reliability (*ω*) of the subscale scores and the total self-compassion score. These showed that the subscales ranged from *ω* .80 to .93 and the scale had an overall *ω* of .96 showing that the subscales were representative of both self-compassion and the six factors. There was greater variance in the ωH indices with scores ranging from .05 (self-kindness) to .46 (isolation). The omega indices for the t1 data echoed these results.

**Table 4:** Reliability indices for the Self-Compassion Scale and variance explained in bi-factor model

Scales	No. of items	Alpha *α*			Omega *ω*		Omega H ωH		M (SD)	
		t1	t2	Retest	t1	t2	t1	t2	t1	t2
SCS overall	26	.92	.95	.93	.94	.96	.84	.90	2.82 (.65)	3.07 (.32)
Self-kindness	5	.82	.89	.87	.89	.93	.08	.05	2.72 (.79)	2.8 (.86)
Common humanity	4	.77	.83	.80	.79	.85	.51	.41	3.03 (.86)	3.09 (.87)
Mindfulness	4	.71	.75	.81	.76	.8	.29	.26	3.17 (.76)	3.23 (.73)
Self-judgement	5	.81	.89	.89	.83	.9	.26	.20	3.33 (.85)	3.27 (.93)
Isolation	4	.77	.80	.83	.78	.81	.51	.46	3.31 (.92)	3.26 (.89)
Over-identification	4	.75	.82	.87	.73	.82	.34	.40	3.37 (.89)	3.29 (.94)

*No. of items* number of items on the factors, *α* Cronbach’s alpha, *ω* coefficient omega, *ωH* omega hierarchical, *M*​ Mean, *SD *standard deviation

As in Neff et al.’s (2017) paper, we calculated the variance in total scores that is explained by the overarching self-compassion factor (ωH/*ω*). In our data, 89% of t1 and 94% of t2 variance in total scores resulted from the overarching self-compassion factor.

## Discussion

The SCS is a widely used measure of self-compassion and its factor structure has received a great deal of research interest. This study provided an independent evaluation of the SCS’s factor structure and replicated the models evaluated by Neff et al. (2017). Specifically, the outcomes of this study echo those found in Neff et al.’s (2017) study, in particular the results from her student sample. We found the SCS to be reasonably reliable with both the overall scale and subscales having relatively high internal reliability and good test-retest reliability. Of the models we investigated, we found that the bi-factorial model consisting of the six-factor correlated model and an overarching self-compassion factor was the best fit to our data. This supports Neff’s (2016) conceptualisation of self-compassion as having six distinct factors that are influenced by a concurrent (self-compassion) factor and the use of the SCS to give both an overall self-compassion score, or to use the scores from individual subscales. The inclusion of a general self-compassion factor accounted for some of the shared variance between factors and improved the model fit across all of our fit criteria (TLI = 0.94, CFI = 0.95) and the AIC suggested that this model was the best fitting of all the models. When we ran the same analyses on the t1 data, the bi-factorial model did not fulfil all of our fit criteria (TLI < 0.9), but remained the closest fit to our data. In Neff et al.’s (2017) recent paper, the bi-factorial model was not as good a fit as the six-factor solution in any of the populations, but it still demonstrated an acceptable fit in all of the populations with the exception of the clinical sample. Van Prooijen and Van Der Kloot (2001), however, emphasised that there is never ‘one single true model’ as data are subject to individual differences.

The omega indices showed further support for the bi-factorial construction of self-compassion as the subscales ranged from *ω* = .80 to .93 and the total score had an *ω* of .96 suggesting that the subscales and the overarching scale are representative of both self-compassion and the six factors. With the inclusion of the overarching self-compassion factor, the ωH indices reduced to between .05 (self-kindness) and .46 (isolation). Lower ωH scores indicate that a greater proportion of that factor’s variance has been explained by the overarching self-compassion factor rather than the individual factor(s). Self-kindness, for instance, appeared to be comprised largely of self-compassion as the variance reduced by 88% (t2) when the overarching factor was included. We also calculated the percentage variance in total scores (89% of t1 and 94% of t2) explained by the overarching self-compassion factor (Table 5). Our findings echo the percentages reported by Neff et al. (2017) who found that the general self-compassion factor accounted for 90–95% of variance across their samples. These omegas indicate that both the scores from the specific factors and from the total score can be interpreted with confidence.

**Table 5:** Factor loadings of SCS items on subscales for six-factor correlated and bi-factorial model using time 1 and time 2 data

Subscale	t2 6-factor	t2 bi-factorial	t1 6-factor	t1 bi-factorial
Self-kindness				
5	.71	.79	.67	.78
12	.82	.86	.78	.85
19	.85	.88	.78	.83
23	.71	.76	.61	.63
26	.83	.85	.65	.66
Self-judgement				
1	.82	.74	.70	.59
8	.76	.71	.69	.58
11	.76	.69	.64	.56
16	.81	.70	.73	.58
21	.75	.72	.66	.60
Common humanity				
3	.70	.53	.62	.39
7	.75	.49	.69	.34
10	.76	.56	.76	.44
15	.75	.63	.65	.51
Isolation				
4	.76	.60	.74	.54
13	.67	.55	.65	.35
18	.59	.42	.58	.28
25	.75	.60	.68	.44
Mindfulness				
9	.55	.39	.52	.33
14	.78	.62	.73	.58
17	.73	.65	.69	.57
22	.58	.61	.57	.60
Over-identification				
2	.81	.62	.77	.54
6	.75	.64	.70	.45
20	.70	.40	.54	.26
24	.64	.44	.58	.31

The six-factor correlated model was also a good fit to our t2 data and the fit was comparable with previous research (Neff 2003a, b: TLI = 0.9, CFI = 0.91. Neff et al., 2017: student sample: TLI = 0.92, CFI = 93. The present study: TLI = 0.92 and CFI = 0.93). In our model, the items loaded well onto the proposed factors with loadings ranging from .55 to .85. These were comparable to those from the student sample in Neff et al.’s recent paper (Neff et al. 2017). Very similar factor loadings were found when the CFA was run on the t1 data. Internal consistency was mostly good within the subscales. However, we found that perceived isolation was highly correlated with the over-identification and self-judgement factors. Correlations of this level (0.94 and 0.90 respectively) can indicate poor discriminant validity between subscales, but in some, as is probable in this case, they can be indicative of a shared latent variable that impacts upon the scale over and above the impact of the factors (Gaskin 2016). The inconsistencies in models found by previous research might suggest the latter may be the case and findings from this study support this conjecture.

The five-factor model from our EFA was not supported during the confirmatory procedures from either of our time points; however, this is not an unusual outcome in cross-validation studies as no parameters are set in EFA and the data are allowed to inform the model formation whereas the CFA procedure is run with more restrictions in place (van Prooijen and van der Kloot 2001). Neither the single-factor nor two-factor models fitted our data. This might suggest that the operationalisation of self-compassion is more complicated than it being a single construct or a sum of the positive and negative items. To address concerns regarding the inclusion of the negative components of self-compassion, some research has adopted the two-factor model to measure self-coldness and self-compassion scores (e.g. Gilbert et al. 2011). Although the fit indices were approaching a fit to the data, this model did not reach acceptable levels of fit; therefore, we found no support for using the SCS in this way. We found similar outcomes for the higher-order model to Neff et al. (2017). In higher-order models, the overarching factor accounts for all variance between the factors that load on to it. It does not allow the factors to retain any individual influence on the model. This does not fit with Neff’s (2016) conceptualisation of self-compassion as consisting of both self-compassion and interrelated components.

The lack of fit for the two-factor model should also alleviate some concerns that the SCS may be affected by the item scoring method (e.g. López et al. 2015, Muris and Petrocchi 2017). The fact that the single compassion factor was not a fit to our data supports Neff et al.’s (2017) ascertainment that although the SCS can be used to yield a total score compassion, it is not constructed of a single dimension.

Self-compassion is an important psychological construct and it is imperative that we advance our understanding of how it is optimally operationalised. Our findings support the view that compassion is a multi-faceted construct that is more complicated than being comprised exclusively of the positive components of self-compassion. Muris and Petrocchi (2017), however, reported greater associations between the negative components and psychopathology than the positive components of the SCS. These authors highlight the importance that the scoring method can have on a scale in that the reverse scored items might serve to inflate the self-compassion score, thus increasing the association between the self-compassion total score and psychopathology. The bi-factorial construct of the SCS affords researchers the opportunity to explore the impact of the individual factors as well as the overall total score and address this concern. In the present study, our inter-factor correlations were stronger between negative components of self-compassion than those between the positive ones. More research needs to be conducted into the mechanisms underlying the components of self-compassion and to explore how much impact each factor has under various circumstances or populations.

## Limitations and Future Directions

The study employed a student sample which was three quarters female. Model fit should be tested across other populations to establish what models of self-compassion are most appropriate in different populations and studies should investigate how gender impacts upon model fit. In future studies, modelling techniques ought to be reflective of the complexity of self-compassion and, as such, assess the presence of a shared compassion factor by using bi-factorial and other in-depth structural equation modelling techniques. Factorial and structural modelling techniques are continuing to develop, and in a recent investigation, Tóth-Király et al. (2016) applied exploratory structural equation modelling techniques to a Hungarian version of the SCS. Applying these techniques to the original language version of the SCS would allow for more rigorous testing of this important construct.

Our study found that the SCS can be used to give subscale totals and to give an overall total compassion score. Despite the scale’s extensive use, however, we have little understanding of how the six components of the SCS interact with each other and more work needs to be done to understand if all the factors contribute equally to a person’s compassion or if one area is potentially more important than another. In this vein, Muris and Petrocchi (2017) also emphasised the need for studies to investigate the predictive value of the different components of the SCS in the aetiology of psychopathology. To facilitate this, research into the SCS needs to move away from cross-sectional studies and employ more prospective designs which would also allow the investigation of the stability of self-compassion over time. Studies could also be designed to determine how self-compassion is affected by the presence of stressful life events, particularly events that increase feelings of self-criticism and failure (e.g. Tóth-Király et al. 2016), and allow exploration of the relationship between these and the latent variables of the SCS.

More research emphasis needs to be placed on the positive components of mental health rather than the negative aspects, and as such, self-compassion is an important area that deserves much more research attention. Thus far, research into self-compassion has primarily focussed on its association with mental health problems such as depression, anxiety and stress; however, how self-compassion is related to more complex mental health problems including experiences such as paranoia and distressing voices, self-harm and suicide is worthwhile. Moreover, the role of self-compassion in recovery merits more attention (Anthony 1993; Leamy et al. 2011). Our research reiterates Neff et al.’s (2017) findings that the SCS can be used as the six-factor and bi-factorial model, thereby further emphasising the complexity of self-compassion. Our findings also support the use of the SCS to give a total score as suggested by Neff and colleagues (Neff 2003a, b, Neff et al. 2017). However, in light of Muris and Petrocchi (2017) recent meta-analysis, further examination of the contributions of the individual factors, particularly the negative factors, to the overall self-compassion score is vital. In sum, further research into this complex construct is needed to establish the impact of the individual components on the models of the SCS and how these components interact within mental health and illness.
